# Positive Airway Pressure Therapies and Oxygen Therapy in Obstructive Sleep Apnea (OSA): 5-Year Survival

**DOI:** 10.3390/jcm14248647

**Published:** 2025-12-06

**Authors:** Juan Sebastian Hernández Puentes, Alirio Rodrigo Bastidas, Eduardo Andres Tuta Quintero, Juan David Acosta Otero, Valeria Leyton Franco, Juan Diego Castro Córdoba, Lina María López Nuñez, Isabella Lenhardt Guaqueta, Alejandra Mora Vega, Paola Stefanny Martínez Sáenz, Charbel Kamil Faizal Gomez, María Catalina Vaca Espinosa, Cristian Felipe Cardona Molina, Gabriela Diaz Romero, Avril Johanna Rubio Noel

**Affiliations:** 1 School of Medicine, Universidad de La Sabana, Km 7, Autonorte de Bogota, Chía 250001, Colombia; 2 Internal Medicine, Universidad de La Sabana, Chía 170001, Colombia; 3 Clinical Medicine Applied Research Group, Universidad de La Sabana, Chía 170001, Colombia; 4 Epidemiology, Universidad de La Sabana, Chía 250001, Colombia

**Keywords:** sleep apnea syndromes, sleep apnea, obstructive, continuous positive airway pressure, oxygen, adherence interventions, survival

## Abstract

**Background:** Obstructive sleep apnea (OSA) is a highly prevalent disorder associated with increased morbidity and mortality. Continuous positive airway pressure (CPAP) remains the first-line therapy, but its long-term effectiveness is limited by suboptimal adherence, with only 50–60% of patients achieving the recommended use. Evidence on adherence with alternative modalities, such as bilevel positive airway pressure (BiPAP) or oxygen therapy, is even more limited. Furthermore, few studies have directly compared these treatments with each other, particularly in relation to survival outcomes. **Objective**: Evaluate 5-year survival in patients with OSA treated with CPAP, BIPAP, or oxygen therapy. **Methods**: A retrospective cohort study with survival analysis was conducted in subjects with OSA followed at a tertiary-level institution in Colombia between January 2005 and December 2021. **Results:** Among 3039 patients with OSA (mean age 59.6 years; 59.8% male), the five-year mortality rate was 5.8%. Deceased patients presented a higher prevalence of comorbidities, including hypertension, diabetes, and cardiovascular disease (all *p* < 0.001). Adherence to CPAP was significantly lower in deceased patients. Survival analysis showed the highest five-year survival among adherent CPAP/Auto-CPAP users (95.6%), followed by non-adherent CPAP (95%) and adherent BiPAP users (94.1%). Lower survival was observed in non-adherent BiPAP users (91.7%) and oxygen therapy patients (80.6%). In multivariable analysis, treatment type, older age, congestive heart failure, chronic lung disease, and metastatic cancer were independently associated with increased mortality risk. **Conclusions**: Five-year survival in patients with obstructive sleep apnea was significantly associated with the treatment modality and adherence level.

## 1. Introduction

Obstructive sleep apnea (OSA) is a chronic respiratory disorder affecting approximately one billion people worldwide, with an estimated prevalence of 10%, a figure that continues to rise, largely driven by the global increase in obesity [1,2]. It is characterized by recurrent episodes of upper airway obstruction during sleep, leading to sleep fragmentation, intermittent hypoxemia, and daytime sleepiness [3]. In addition to impaired quality of life, OSA is also associated with an increased risk of mortality. Therapeutic management is based on positive airway pressure devices, selected surgical interventions, and lifestyle changes, with continuous positive airway pressure (CPAP) being the most commonly used treatment [4].

In the current literature, most randomized controlled trials have evaluated CPAP versus placebo, sham CPAP, or educational interventions, with follow-up periods ranging from six months to five years. While these studies have shown improvements in parameters such as the apnea–hypopnea index (AHI) and daytime sleepiness, assessment of their impact on mortality and other clinical outcomes remains limited. Additionally, there is notable methodological heterogeneity and a lack of direct comparative studies among different therapeutic modalities, such as CPAP, bilevel positive airway pressure (BiPAP), and oxygen therapy [5,6]. Although CPAP has shown efficacy in patients without respiratory comorbidities, BiPAP is usually reserved for patients with hypoventilation or CPAP intolerance, with no evidence of superiority in isolated OSA [7]. Oxygen therapy improves nocturnal saturation but does not reduce AHI or sleepiness [8]. Despite its widespread use, current studies do not systematically integrate long-term comparisons between these strategies, nor do they adequately account for key clinical factors such as therapy adherence [9].

Low adherence to positive airway pressure therapy remains one of the main barriers to achieving effective control of OSA. Adherence is defined as device use ≥4 h per night on at least 70% of days; however, only 50–60% of patients reach this minimum threshold in the long term [10,11]. Despite their importance, few studies have comprehensively compared different therapeutic modalities while simultaneously considering adherence and survival. In this context, the objective of this study was to evaluate the 5-year survival of patients with OSA treated with CPAP, BIPAP, or oxygen therapy.

## 2. Materials and Methods

A retrospective cohort study with survival analysis was conducted in subjects with OSA who were followed at a tertiary-level institution in Colombia between January 2005 and December 2021.

### 2.1. Eligibility Criteria

Subjects included were adults over 18 years old with a diagnosis of OSA, defined by an apnea–hypopnea index (AHI) greater than 5 events per hour of sleep, confirmed by polysomnography (PSG) following the criteria of the American Academy of Sleep Medicine (AASM) [12]. Eligible patients presented symptoms such as chronic snoring, excessive daytime sleepiness, persistent fatigue, and sleep disturbances. Exclusion criteria included other sleep disorders or medical conditions with similar symptoms, patients without follow-up survival data, incomplete AHI information, and those without treatment data.

### 2.2. Variables

Data were collected on demographic characteristics, comorbidities, vital signs, physical examination, evolution of clinical symptoms at admission, and type of treatment (CPAP, BiPAP, Oxygen). Adherence was defined as device use ≥4 h per night on at least 70% of days; data were obtained from device downloads longitudinally over time. The outcome variable was the 5-year overall survival.

To reduce transcription bias, data were verified by at least two members of the research team directly from the electronic medical records, and personnel collecting the information were trained for this task.

### 2.3. Sample Size

The sample size was calculated using the method described by Ahnn and Anderson [13], based on the log-rank test to compare survival curves. Previous data from a study by Pépin et al. [14] were used, where the mortality was 2.3% in patients with CPAP and 3.6% in those who discontinued CPAP therapy, with a sample size ratio of 3:1. Assuming a 95% confidence level, 90% power, and an estimated 10% loss, a minimum of 3001 subjects were determined.

### 2.4. Data Analysis

Data were extracted directly from electronic medical records, thoroughly reviewed, and imported into the Research Electronic Data Capture (REDCap) system. They were then exported to Excel for final analysis using Stata 14 software. Qualitative variables were summarized by count and percentage; quantitative variables were expressed as mean and standard deviation if normally distributed, or median and interquartile range if not. Normality was assessed using the Kolmogorov–Smirnov test. For independent sample comparisons, the two-sample *t*-test with Welch correction or the Mann–Whitney U test was used as appropriate.

Survival analysis was conducted using the Kaplan–Meier method to estimate the survival curves for patients with OSA under different therapies. Differences between the curves were evaluated using the log-rank test. Multivariate analysis was performed using Cox proportional hazards regression to identify potential confounders for 5-year mortality, adjusting for relevant demographic and clinical variables with corresponding hazard ratios (HRs) and 95% confidence intervals. Statistical significance was set at *p* < 0.05.

## 3. Results

### 3.1. General Characteristics of the Population

**Figure 1:**
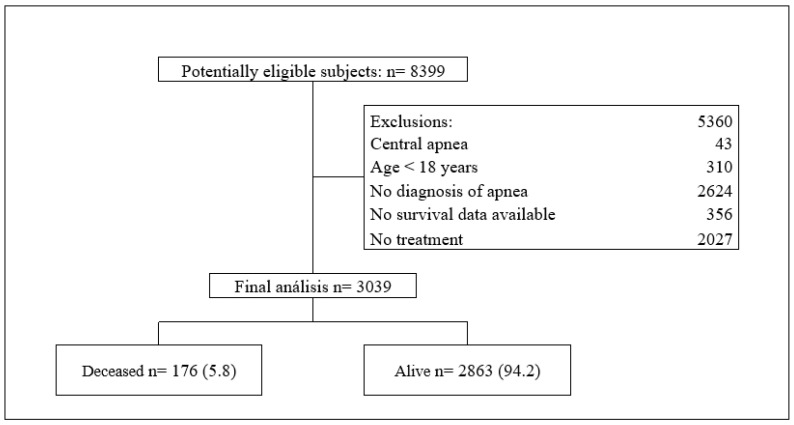
Flowchart of study.

A total of 3039 patients with obstructive sleep apnea were included (Figure 1), with a mean age of 59.6 years (SD ± 13.93) and a predominance of males (59.8%). The five-year mortality rate was 5.8%, occurring more frequently in individuals aged >65 years (75.6% vs. 34.7%; *p* < 0.001). The most common symptoms were snoring (53.2%), daytime hypersomnolence (43.4%), and insomnia (29.3%) (Table 1).

### 3.2. Medical History and Comorbidities of the Population

The most common comorbidities were hypertension (61.7%), smoking (40.3%), and diabetes mellitus (21.7%), all of which were more prevalent among the deceased patients (*p* < 0.001). Higher rates of cardiovascular disease, dementia (19.3% vs. 4.1%; *p* < 0.001), chronic lung disease (60.2% vs. 20.4%; *p* < 0.001), and connective tissue disease (13.1% vs. 6.7%; *p* = 0.001) were also observed in this group. Uncomplicated and complicated diabetes, renal failure, and neurological sequelae from stroke were more frequent among deceased patients (*p* < 0.001). Cancer (24%, *p* < 0.001) and metastasis (10.5% vs. 0.7%, *p* < 0.001) were also associated with higher mortality rates (Table 2).

### 3.3. Treatment Adherence

Regarding adherence to CPAP therapy, deceased patients had a lower proportion of days of use (60.2 vs. 69.9%, *p* < 0.001) and a lower overall adherence rate (66.9% vs. 78%; *p* < 0.001). The proportion of days with more than four hours of use was lower among deceased patients, although this difference was not statistically significant (62.1% vs. 69.2%; *p* = 0.067) (Table 1).

### 3.4. Survival Analysis

**Figure 2:**
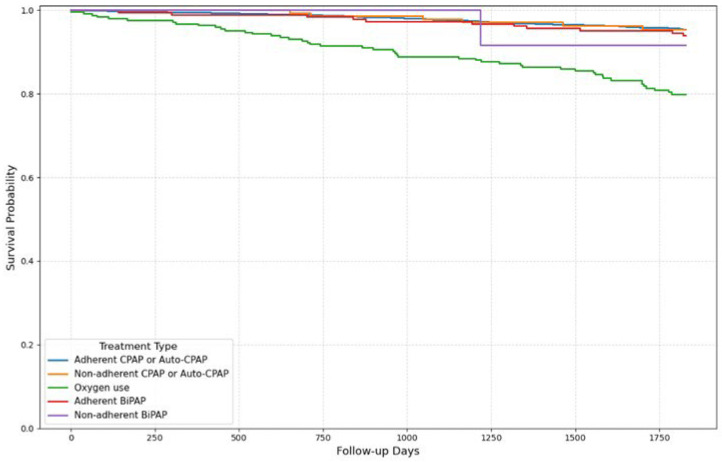
Survival curve Kaplan–Meier by treatment type.

Five-year survival varied significantly (*p* < 0.001) according to the type of treatment, as illustrated by the Kaplan–Meier survival curve (Figure 2). Patients adherent to CPAP or Auto-CPAP had the highest survival rate (95.6%), followed by non-adherent CPAP users (95%), and adherent BIPAP users (94.1%). In contrast, survival was lower among nonadherent BIPAP users (91.7%) and those using supplemental oxygen (80.6%) (Table 3).

In multivariable Cox regression analysis, treatment type was independently associated with mortality risk (HR, 1.20; 95% CI, 1.03–1.40; *p* = 0.018). Other variables associated with increased mortality included age (HR: 1.05 per additional year; 95% CI: 1.03–1.07; *p* < 0.001), congestive heart failure (HR: 2.20; 95% CI: 1.46–3.30; *p* < 0.001), chronic lung disease (HR: 1.90; 95% CI: 1.27–2.86; *p* = 0.002), and metastatic cancer (HR: 5.18; 95% CI: 2.58–10.40; *p* < 0.001) (Table 4).

## 4. Discussion

In the present study, five-year survival in patients with obstructive sleep apnea (OSA) Treatment modality and adherence were associated with differences in 5-year survival (HR: 1.20; 95% CI: 1.03 to 1.40; *p* = 0.018). Patients who adhered to CPAP or Auto-CPAP had the highest survival rates (95.6%), followed by those who did not meet the adherence criteria and adherent BIPAP users. Lower survival rates were observed among non-adherent BIPAP users and those who used oxygen (80.6%). Deceased patients were older and had a higher prevalence of comorbidities, such as hypertension, diabetes, chronic lung disease, heart failure, and metastatic cancer. Additionally, they showed lower overall adherence to CPAP therapy and a lower proportion of days of effective use.

The analysis of outcomes according to different therapeutic strategies showed that both treatment modality and specific comorbidities are associated with variations in mid-term survival in patients with OSA. As a local Colombian study, these findings provide valuable insights into the relevance of these associations within our specific healthcare and population context. Patients with sustained adherence to CPAP or auto-CPAP showed higher survival rates, a finding related to the reduction in the apnea–hypopnea index and the frequency of nocturnal desaturations, parameters linked to a lower incidence of cardiovascular events, and mortality [5,15]. In the subgroup treated with BIPAP, the survival was slightly lower than that with CPAP, regardless of adherence. This may be due to the fact that BIPAP is usually indicated in patients with hypoventilation or more advanced chronic respiratory diseases, who more frequently present with heart failure, chronic lung disease, and renal failure, translating into a higher comorbidity burden and less favorable baseline prognosis compared to the CPAP group [6]. The exclusive use of supplemental oxygen was associated with lower survival, consistent with evidence indicating that this strategy does not directly address airway obstruction or modify exposure to intermittent hypoxemia, which are factors influencing the clinical course of OSA [8].

Regarding adherence, deceased patients showed a lower proportion of CPAP use days and a significantly lower adherence rate than the rest of the cohort. Although the difference in the percentage of nights with effective use greater than four hours did not reach statistical significance, the observed trend aligns with previous data, suggesting a direct relationship between therapeutic compliance and reduction in adverse events [16]. Furthermore, the presence of comorbidities such as congestive heart failure, chronic obstructive pulmonary disease (COPD), renal failure, and metastatic cancer among deceased patients underscores the importance of considering the overall clinical profile when selecting the treatment modality. Tailored strategies aimed at optimizing adherence should be prioritized, as recommended by the American Academy of Sleep Medicine guidelines [5].

In terms of population characteristics, a predominance of male patients and a mean age of approximately 60 years were observed, which is consistent with the results of previous studies [2,17]. Mortality was higher among patients over 65 years of age, who also had a lower body mass index and higher residual apnea–hypopnea index, suggesting reduced physiological reserve. Although some studies have proposed a greater impact of OSA in younger adults, this relationship has not yet been clearly established [18]. In the Colombian context, altitude conditions could exacerbate intermittent hypoxemia, promote pulmonary vascular remodeling, and reduce physiological reserves. As noted by Brito et al., chronic or intermittent hypobaric hypoxia, typical of certain South American regions, may intensify hypoxic stress and worsen outcomes in individuals with lower adaptive capacities, such as older adults [19]. Deceased patients also exhibited lower frequencies of typical symptoms, such as snoring and lower CPAP adherence, which in older adults may reflect lower disease perception and delayed access to treatment.

Comorbidities play a key role in patient survival. Cardiovascular, respiratory, and oncologic conditions are more common among deceased patients with congestive heart failure, COPD, complicated diabetes, and metastatic cancer, which are factors associated with a worse prognosis [20,21,22]. These conditions not only reflect greater systemic deterioration but may also amplify the effects of OSA. In this context, OSA acts as a destabilizing factor, and its management should comprehensively consider the patient’s clinical profile to optimize both adherence and clinical outcomes [23,24].

The limitations of this study include its observational design and the use of clinical records as the primary source of information, which may have led to omissions or variability in data quality. Although standardized protocols were applied to minimize information bias, its complete elimination cannot be guaranteed. Furthermore, as this was a single-center study, the generalizability of the findings to other populations may be limited. Another important limitation is the potential influence of unmeasured confounders, such as differences in socioeconomic status, medication use, or treatment duration, which could have affected the observed associations. Therefore, these results should be interpreted with caution and further validated in larger, multicenter studies. Nonetheless, the adequate sample size and the consistency of the results support the internal validity of the analysis. Future multicenter research could provide a more comprehensive understanding of the impact of adherence and comorbidities on the survival of patients with OSA.

## 5. Conclusions

Five-year survival in patients with obstructive sleep apnea was significantly associated with the treatment modality and adherence level. The presence of severe comorbidities and lower CPAP adherence were related to higher mortality.

## Figures and Tables

**Table 1:** General characteristics of the population.

	Total Population n = 3039	Alive n = 2863	Deceased n = 176	*p*-Value
Age (years) x (sd)	59.6 (13.93)	58.8 (13.63)	72.2 (12.72)	<0.001
>65 years n (%)	1126 (37.1)	993 (34.7)	133 (75.6)	<0.001
Male n (%)	1818 (59.8)	1712 (59.8)	106 (60.2)	0.910
**Anthropometric measurements**				
Weight Kg x (sd)	80 (15.67)	80.3 (15.64)	75.6 (15.62)	<0.001
Height Mt’s x (sd)	1.63 (0.1)	1.64 (0.1)	1.61 (0.09)	<0.001
BMI Kg/m^2^ x (sd)	30 (5.34)	30 (5.28)	29 (6.14)	0.039
**Symptoms**				
Daytime hypersomnia n (%)	1126 (43.4)	1068 (43.8)	58 (37.2)	0.104
Insomnia n (%)	751 (29.3)	709 (29.5)	42 (27.1)	0.530
Snoring n (%)	1384 (53.2)	1317 (53.9)	67 (42.9)	0.008
Tired-fatigued n (%)	765 (29.8)	722 (30)	43 (27.6)	0.522
Observed_Apneas n (%)	1063 (42.1)	998 (42.1)	65 (42.2)	0.974
Neck diameter x (sd)	41.3 (5.14)	41.3 (5.17)	40.9 (4.35)	0.194
**CPAP Adherence**				
Days of CPAP use x (sd)	69.5 (20.13)	69.9 (19.84)	60.2 (26.09)	<0.001
CPAP Adherence % x (sd)	77.6 (21.82)	78 (21.47)	66.9 (28.99)	<0.001
Days of use greater than 4 h x (sd)	68.6 (29.92)	69.2 (28.09)	62.1 (50.79)	0.067
Average hours of CPAP use M (IQR)	5.2 (4–6.53)	5.5 (3.7–7.58)	5.2 (4–6.5)	0.992
Residual AHI M (IQR)	8.9 (3–27.8)	15 (4.05–29.3)	8.5 (3–27.6)	0.110

Sd: standard deviation; M: median; IQR: interquartile range; BMI: body mass index; CPAP: continuous positive airway pressure.

**Table 2:** Medical history of the population.

	Total Population n = 3039	Alive n = 2863	Deceased n = 176	*p*-Value
**Medical History n (%)**				
Smoking	1222 (40.3)	1123 (39.3)	99 (56.3)	<0.001
Hypertension	1875 (61.7)	1728 (60.4)	147 (83.5)	<0.001
Depression	298 (9.8)	276 (9.6)	22 (12.5)	0.217
Alcohol use	170 (5.6)	147 (5.1)	23 (13.1)	<0.001
Myocardial infarction	339 (11.2)	301 (10.5)	38 (21.6)	<0.001
Atrial fibrillation	218 (7.2)	176 (6.2)	42 (23.9)	<0.001
Congestive heart failure	355 (11.7)	286 (10)	69 (39.2)	<0.001
Peripheral vascular disease	190 (6.3)	169 (5.9)	21 (11.9)	0.001
Cerebrovascular disease	229 (7.5)	194 (6.8)	35 (19.9)	<0.001
Dementia	151 (5)	117 (4.1)	34 (19.3)	<0.001
Chronic lung disease	689 (22.7)	583 (20.4)	106 (60.2)	<0.001
Connective tissue disease	214 (7)	191 (6.7)	23 (13.1)	0.001
Peptic ulcer	354 (11.7)	330 (11.5)	24 (13.6)	0.399
Non-malignant liver disease	265 (8.7)	249 (8.7)	16 (9.1)	0.900
Liver disease	67 (2.2)	60 (2.1)	7 (4)	0.099
Diabetes	659 (21.7)	586 (20.5)	73 (41.5)	<0.001
Diabetes with end-organ damage	89 (3.1)	73 (2.7)	16 (9.4)	<0.001
Hemiplegia/stroke sequelae	88 (3.1)	73 (2.7)	15 (8.8)	<0.001
Renal failure	216 (7.1)	177 (6.2)	39 (22.2)	<0.001
Leukemia	8 (0.3)	7 (0.2)	1 (0.6)	0.416
Lymphoma	14 (0.5)	12 (0.4)	2 (1.1)	0.173
Other types of cancer	263 (8.7)	221 (7.7)	42 (24)	<0.001
Metastasis	37 (1.3)	19 (0.7)	18 (10.5)	<0.001
HIV/AIDS	5 (0.2)	4 (0.1)	1 (0.6)	0.174
Traffic accidents	119 (3.9)	107 (3.7)	12 (6.8)	0.041

HIV: human immunodeficiency virus; AIDS: acquired immune deficiency syndrome.

**Table 3:** Type of treatment.

	Total Population	Alive	Deceased	*p*-Value
Adherent CPAP or Auto-CPAP n (%)	2454	2345 (95.6)	109 (4.4)	<0.001
Non-adherent CPAP or Auto-CPAP n (%)	140	133 (95)	7 (5)	
Adherent BiPAP n (%)	186	175 (94.1)	11 (5.9)	
Non-Adherent BiPAP n (%)	12	11 (91.7)	1 (8.3)	
Oxygen Use n (%)	247	199 (80.6)	48 (19.4)	

CPAP: continuous positive airway pressure; BiPAP: bilevel positive airway pressure.

**Table 4:** Cox regression: independent characteristics associated with mortality.

	Hazard Ratio	95% Confidence Interval (CI)		*p*-Value
Positive pressure treatment	1.204	1.032	1.404	0.018
Age (years)	1.051	1.032	1.070	<0.001
Sex	1.120	0.761	1.647	0.565
Body mass index	0.981	0.946	1.017	0.289
Smoking history	0.990	0.686	1.427	0.955
Hypertension	1.114	0.657	1.889	0.690
Alcohol use	1.423	0.825	2.456	0.205
Myocardial infarction	1.035	0.684	1.566	0.871
Atrial fibrillation	1.208	0.783	1.863	0.392
Congestive heart failure	2.198	1.463	3.302	<0.001
Peripheral vascular disease	0.984	0.590	1.639	0.950
Cerebrovascular disease	1.125	0.703	1.801	0.623
Dementia	1.265	0.780	2.052	0.340
Chronic lung disease	1.904	1.269	2.856	0.002
Connective tissue disease	1.597	0.950	2.684	0.077
Diabetes	1.370	0.942	1.993	0.099
Chronic kidney disease	1.425	0.934	2.173	0.100
Other cancer	1.097	0.676	1.778	0.708
Metastatic cancer	5.181	2.582	10.396	<0.001

## Data Availability

We confirm that the dataset underlying this study is held and managed by the Clinical Medicine Applied Research Group (Code: COL0084256), affiliated with the Universidad de La Sabana. The extraction, use, and management of these data were explicitly authorized by the institutional Research Ethics Committee, which approved the study protocol and designated this research group as the responsible entity for data handling and custody. The group is committed to ensuring secure and confidential data management and evaluating any external data access requests in accordance with institutional, legal, and ethical standards. More information about the group can be found on the official MinCiencias GRUPLAC platform (https://scienti.minciencias.gov.co/gruplac/jsp/visualiza/visualizagr.jsp?nro=00000000007713 (accessed on 10 September 2025)). At present, the group’s director is Alirio Rodrigo Bastidas Goyes, who may be contacted at alirio.bastidas@unisabana.edu.co for data access inquiries. In case of any changes in leadership or contact information, the GRUPLAC page will be updated to ensure persistent accessibility.

## Abbreviations

The following abbreviations are used in this manuscript:

OSA	Obstructive Sleep Apnea
CPAP	Continuous Positive Airway Pressure
Auto-CPAP	Automatic Continuous Positive Airway Pressure
BiPAP	Bilevel Positive Airway Pressure
AHI	Apnea–Hypopnea Index
PSG	Polysomnography
AASM	American Academy of Sleep Medicine
HR	Hazard Ratio
CI	Confidence Interval
HIV	Human Immunodeficiency Virus
AIDS	Acquired Immune Deficiency Syndrome
Sd	Standard Deviation
M	Median
IQR	Interquartile Range
BMI	Body Mass Index
COPD	Chronic Obstructive Pulmonary Disease
REDCap	Research Electronic Data Capture
